# Correction to Pharmacokinetics and pharmacodynamics of itepekimab in adults with moderate‐to‐severe atopic dermatitis: Results from two terminated phase II trials

**DOI:** 10.1111/cts.70026

**Published:** 2024-09-19

Kosloski MP, Guttman‐Yassky E, Cork MJ, et al. Pharmacokinetics and pharmacodynamics of itepekimab in adults with moderate‐to‐severe atopic dermatitis: Results from two terminated phase II trials. *Clin Transl Sci*. 2024; 17: e13874. doi:10.1111/cts.13874

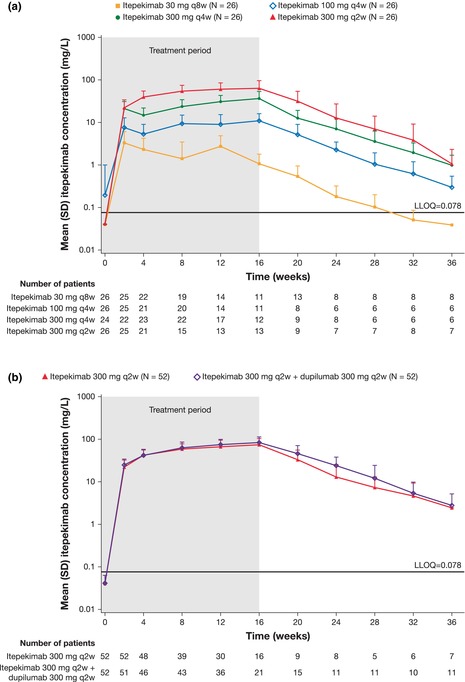

In Figure 2 panels a and b, the Y‐axis label should be mg/L, not mg/mL.

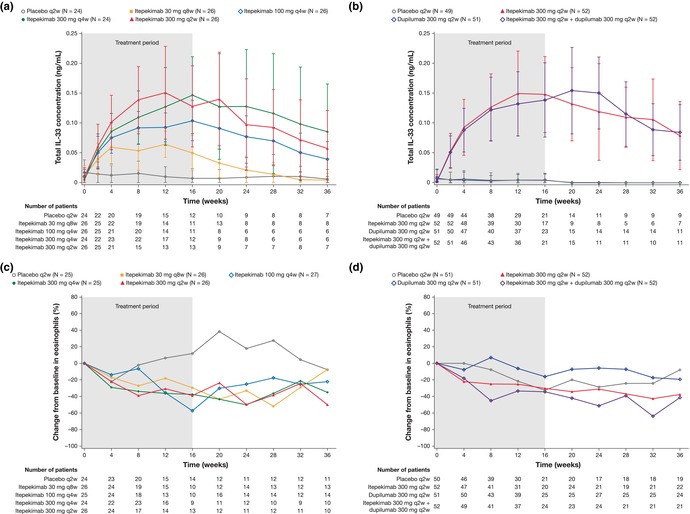

In Figure 3 panels a and b, the Y‐axis label should be ng/mL, not mg/L.

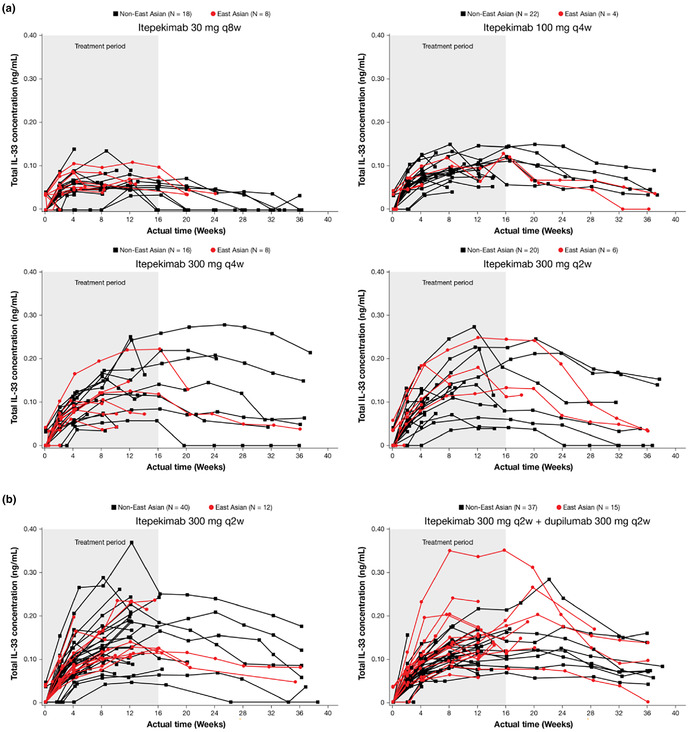

Concentration time proles of total IL‐33 in East Asian and non‐East Asian patients during the study period in the (A) dose‐ranging study and (B) proof‐of‐concept study.

In Figure S4 (all panels), the Y‐axis label should be ng/mL, not mg/L.

We apologize for this error.

